# Decrease in cholesterol levels during the immunotherapy of cancer with interleukin-2.

**DOI:** 10.1038/bjc.1991.434

**Published:** 1991-11

**Authors:** P. Lissoni, F. Brivio, S. Pittalis, M. S. Perego, A. Ardizzoia, O. Mauri, S. Barni, S. Crispino, G. Tancini

**Affiliations:** Divisione di Radioterapia Oncologica, Hospital of Monza, Italy.

## Abstract

IL-2, in addition to its immunomodulating and antitumour properties, induces important systemic actions, including cardiovascular, neuroendocrine and metabolic effects. The present study was carried out to evaluate IL-2 effects on cholesterol metabolism. The study included 14 advanced cancer patients (renal carcinoma: ten; colon carcinoma: four), who received IL-2 subcutaneously at a dose of 1.8 x 10(6) IU ml-2 twice daily for 5 days/week for 6 weeks. Venous blood samples were collected 7 days before, on days 0, 3, 7, 14, 21, 42 of IL-2 therapy, and on days 14 and 28 of the rest-period. IL-2 induced a rapid and evident decrease in cholesterol levels, with a normalisation of its concentrations within 7 days in 10/10 hypercholesterolemic patients. The lowest mean levels of cholesterol were reached within the first 2 weeks; after that they still slowly increased. LDL-/HDL-cholesterol ratio was significantly reduced by IL-2 therapy. Cholesterol fall was associated with a marked increase in conjugated biliary acid levels. Finally, triglyceride values increased during IL-2 therapy, but not in a significant manner. These results, by showing that IL-2 exerts an evident and very rapid cholesterol-lowering activity, would represent a further demonstration of the physiological importance of cytokines in the control of cholesterol metabolism.


Br. J. Cancer (1991), 64, 956-958 © Macmillan Press Ltd., 1991

### Decrease in cholesterol levels during the immunotherapy of cancer with Interleukin-2

P. Lissoni[1], F. Brivio[2], S. Pittalis[1], M.S. Perego[1], A. Ardizzoia[1], O. Mauri[3], S. Barni[1], S. Crispino[1] & G. Tancini[1]

[1]*Divisione di Radioterapia Oncologica,* [2]*Divisione di Chirurgia II, Hospital of Monza, 20052 Monza;* [3]*Divisione di Cardiologia, San Raffaele Hospital, Milan, Italy.*

**Summary** IL-2, in addition to its immunomodulating and antitumour properties, induces important systemic actions, including cardiovascular, neuroendocrine and metabolic effects. The present study was carried out to evaluate IL-2 effects on cholesterol metabolism. The study included 14 advanced cancer patients (renal carcinoma: ten; colon carcinoma: four), who received IL-2 subcutaneously at a dose of $1.8 \times 10^6$ IU $ml^{-2}$ twice daily for 5 days/week for 6 weeks. Venous blood samples were collected 7 days before, on days 0, 3, 7, 14, 21, 42 of IL-2 therapy, and on days 14 and 28 of the rest-period. IL-2 induced a rapid and evident decrease in cholesterol levels, with a normalisation of its concentrations within 7 days in 10/10 hypercholesterolemic patients. The lowest mean levels of cholesterol were reached within the first 2 weeks; after that they still slowly increased. LDL-/HDL-cholesterol ratio was significantly reduced by IL-2 therapy. Cholesterol fall was associated with a marked increase in conjugated biliary acid levels. Finally, triglyceride values increased during IL-2 therapy, but not in a significant manner. These results, by showing that IL-2 exerts an evident and very rapid cholesterol-lowering activity, would represent a further demonstration of the physiological importance of cytokines in the control of cholesterol metabolism.

The administration of interleukin-2 (IL-2) in the immunotherapy of cancer may induce important cardiovascular complications, including a severe hypotension, an increased capillary permeability, and cardiac ischaemic disorders (Rosenberg *et al.*, 1987; Lee *et al.*, 1989). These side-effects are less evident when IL-2 is subcutaneously given (Atzpodien *et al.*, 1990). Hepatic, renal and haematological toxicities have been also observed during IL-2 immunotherapy (Rosenberg *et al.*, 1987). Moreover IL-2, as well as other cytokines, in addition to its immunomodulating properties, may also induce important endocrine and metabolic effects (Denicoff *et al.*, 1989; Chambrier *et al.*, 1990). Among the metabolic effects of cytokines, granulocyte-macrophage colony-stimulating factor (GM-CSF) has been shown to reduce cholesterol levels (Nimer *et al.*, 1988). Moreover, tumour necrosis factor (TNF) has appeared to cause hypertriglyceridemia (Sherman *et al.*, 1988). On the contrary, only few data are available up to now about the possible influence of IL-2 on lipid metabolism. Preliminary results have shown a reversible and acute hypocholesterolemia during cancer immunotherapy with high-dose intravenous IL-2 (Wilson *et al.*, 1989). However, the important toxicity of high-dose intravenous IL-2 excludes the possible investigation of IL-2 efficacy in the treatment of cholesterol metabolism disorders. The present study was performed to evaluate the effect of low-dose subcutaneous IL-2 on cholesterol levels and on its fractions in patients with advanced solid neoplasms.

### Materials and methods

Between March 1990 and January 1991, a total of 14 consecutive advanced cancer patients (M/F: 11/3; median age 56 years, range 24-72), followed at San Gerardo Hospital of Monza, entered the study to be treated with IL-2. Ten patients were affected by renal adenocarcinoma, and the remaining four cases by colon adenocarcinoma. Human recombinant IL-2 was supplied by Euro-Cetus (Amsterdam-Holland). IL-2 was subcutaneously injected into different parts of the abdominal wall. The treatment protocol consisted of a 2-day IL-2 pulse of $9 \times 10^6$ IU $m^{-2}$ twice daily, followed by 6 weeks of IL-2 at $1.8 \times 10^6$ IU $m^{-2}$ every 12 h for 5 days/week, corresponding to one IL-2 subcutaneous cycle. Cycles were repeated after a rest period of 4 weeks.

Serum cholesterol, HDL-cholesterol, LDL-cholesterol and triglyceride levels were measured in each patient on venous blood samples collected at 8.00 am, after an overnight fast. Samples were collected 7 days before the start of the immunotherapy, on the same day of the first IL-2 injection, on days 3, 7, 14, 21 and 42 of IL-2 cycle, and on days 14 and 28 of rest period. Moreover, in six patients we have also measured serum levels of conjugated biliary acids by collecting blood samples before IL-2, and on days 3 and 7 of the cycle. Patients followed a dietary regimen consisting of 35 KCal/kg/ideal body weight/day. No patient received drugs influencing cholesterol metabolism during the study. Routinary laboratory tests were repeated every week.

Cholesterol and triglyceride serum levels were measured with a colorimetric method, by using commercial kits (Poli-Industria Chimica, Milan-Italy). HDL- and LDL-cholesterol levels were also determined with a colorimetric method, after lipoprotein precipitation with phosphotungstic acid and magnesium chloride by using commercial kits (Behring, Germany). Finally, serum levels of conjugated biliary acids were measured by RIA, by using commercially available kits (Becton-Dickinson, Orangeburg, NY). The normal values obtained in our laboratory in 100 age- and sex-matched healthy subjects (median age 55 years; range 30-60) and expressed as 95% confidence limits were the following ones: cholesterol: 110-200 mg $dl^{-1}$; HDL: 35-80 mg $dl^{-1}$; LDL: 60-160 mg $dl^{-1}$; triglycerides: 70-160 mg $dl^{-1}$; conjugated biliary acids: 0.6-6 $\mu$mol $l^{-1}$.

Data are shown as mean ± s.e. Results were analysed by the Student's *t*-test, and analysis of variance according to Newman Keuls test and adjusted for a correction factor.

### Results

No ischaemic cardiac toxicity was seen during IL-2 subcutaneous therapy. No important emesis or anorexia occurred during the treatment, and no change in dietary regimen was observed. No patient had changes in body weight greater than 2% during IL-2 cycle. Finally, no important anaemia requiring blood transfusions was seen during the study. An increase greater than 100% in gamma-GT and a mild rise of transaminases were seen in all patients, whereas no signi-

Correspondence: P. Lissoni.
Received 20 March 1991; and in revised form 20 June 1991.

ficant change in total bilirubin mean concentrations was observed during the immunotherapy with IL-2.

Individual values of cholesterol, HDL-, and LDL-cholesterol, found during IL-2 cycle, are reported in Table I, while their mean concentrations are illustrated in Figure 1. Abnormally high levels of cholesterol were seen in 10/14 patients before the start of IL-2 immunotherapy. Cholesterol concentrations fell rapidly with the start of IL-2 administration, with a normalisation of its values in 10/10 hypercholesterolemic patients within 7 days from the beginning of IL-2 therapy. The maximum inhibitory effect on cholesterol levels was obtained within the first 2 weeks of IL-2 therapy; after that, no further decrease was obtained. Mean concentrations of cholesterol significantly decreased during IL-2 treatment, with the lowest levels on day 7 ($P<0.001$ *vs* before). Despite IL-2 administration, cholesterol levels slowly increased after the second week of therapy, and their mean levels found on day 42 were not significantly different from those seen before. Cholesterol rapidly increased in all patients at IL-2 interruption, and its concentrations became substantially similar to those seen before within the 28th day of rest period. Both HDL- and LDL-cholesterol significantly decreased during IL-2 therapy, with a pattern similar to the one showed by the total cholesterol. LDL-/HDL-cholesterol mean ratio, observed during IL-2 therapy, was lower than that seen before, with a difference statistically significant ($2.9 \pm 0.2$ *vs* $3.8 \pm 0.3$; $P = 0.01$). Triglycerides means concentrations progressively increased during IL-2 administration, without, however, any significant difference in respect to those seen before. The peak in triglycerides levels was delayed in respect to the fall in cholesterol values (see Figure 1). Finally the mean levels of conjugated biliary acids significantly increased in response to IL-2 injection, with a peak on day 3 (before: $4.2 \pm 0.9$ μmol $l^{-1}$; after: $15.2 \pm 2.5$ μmol $l^{-1}$; $P<0.005$).

**Figure 1** Serum levels (mean ± s.e.) of cholesterol, HDL-, LDL-cholesterol and triglycerides before and during IL-2 subcutaneous therapy in 14 advanced cancer patients.

### Discussion

The results of this study show that the subcutaneous administration of low-dose IL-2 is able to normalise cholesterol levels in hypercholesterolemic cancer patients. Therefore, this study confirms also with low-dose IL-2 subcutaneous therapy the results previously described by other authors (Wilson *et al.*, 1989) with very high doses of IL-2 given intravenously. The IL-2 cholesterol-lowering activity seems to be more rapid and pronounced than that of 3-hydroxyl-3-methylglutaril-coenzyme A (HMG-CoA) reductase inhibitors (Tobert, 1987), and comparable to that observed with GM-CSF (Nimer *et al.*, 1988). HDL- and LDL-cholesterol are both involved in IL-2-induced fall in total cholesterol concentrations, even though LDL-/HDL-cholesterol ratio decreases during IL-2 subcutaneous therapy, with a following improvement in terms of protection against coronary heart disease (Gorden *et al.*, 1979). Moreover, this study shows that the decrease in cholesterol levels during IL-2 therapy is associated with an increase in triglyceride concentrations. The IL-2-induced TNF rise (Nedwin *et al.*, 1985) would explain triglyceride increase, but not the fall in cholesterol, which does not seem to be influenced by TNF (Sherman *et al.*, 1988). Moreover, at present it is unknown whether IL-2 per se may be responsible for cholesterol-lowering activity, or whether this effect may depend on other cytokines produced in response to IL-2, particularly GM-CSF itself, which secretion is stimulated by IL-2 (Michalevicz *et al.*, 1988). The evidence of a concomitant decrease in HLD- and LDL-cholesterol would suggest an inhibition of cholesterol synthesis, whereas we can exclude that cholesterol fall may be simply due to changes in body weight or in the dietary regimen, since no patient showed important nausea, anorexia or body weight variations during IL-2 therapy. The decline in cholesterol synthesis could be due to a direct inhibition of HMG-CoA reductase, or to a stimulation of LDL and scavenger macrophage receptors, which play a main role in the removal of cholesterol and in the formation of foam cells (Goldstein *et al.*, 1979). In fact, activated T lymphocyte products, which contain IL-2, have been shown either to inhibit HMG-CoA reductase activity (Fogelman *et al.*, 1982), or to modulate LDL macrophage receptor expression (Fogelman *et al.*, 1983). Therefore, we cannot exclude a direct action of IL-2 on hepatic macrophages, which express IL-2 receptors (Malkovsky & Sondel, 1987) and release factors acting on cholesterol metabolism (Cai *et al.*, 1988). However, the IL-2-induced hepatic function damage, as documented by the increase in transaminases and in gamma-GT, could also play a role in determining a reduced cholesterol synthesis, whereas

**Table I** Clinical data and individual serum levels (mg dl) of cholesterol (C), HDL-cholesterol (H), LDL-cholesterol (L) and LDL-/HDL-cholesterol ratio (R) before and during IL-2 subcutaneous therapy in 14 advanced cancer patients

| | | | | *Prior to study* | | | | *Minimum on study* | | | | *Cholesterol* |
|---|---|---|---|---|---|---|---|---|---|---|---|---|
| *Patient* | *Sex* | *Age* | *Tumour histotype* | *C* | *H* | *L* | *R* | *C* | *H* | *L* | *R* | *reduction %* |
| 1 | M | 59 | Renal cancer | 240 | 22 | 118 | 5.4 | 163 | 13 | 48 | 3.7 | 32 |
| 2 | M | 24 | Renal cancer | 181 | 47 | 122 | 2.6 | 159 | 18 | 37 | 2.1 | 17 |
| 3 | M | 67 | Renal cancer | 241 | 40 | 151 | 3.8 | 151 | 21 | 61 | 2.9 | 37 |
| 4 | M | 59 | Renal cancer | 248 | 33 | 159 | 4.8 | 89 | 16 | 66 | 3.9 | 64 |
| 5 | F | 68 | Renal cancer | 258 | 39 | 185 | 4.7 | 110 | 20 | 49 | 2.5 | 57 |
| 6 | M | 49 | Renal cancer | 153 | 44 | 104 | 2.4 | 91 | 24 | 41 | 1.7 | 62 |
| 7 | M | 58 | Renal cancer | 233 | 31 | 157 | 5.1 | 154 | 18 | 71 | 3.9 | 34 |
| 8 | F | 52 | Renal cancer | 158 | 43 | 101 | 2.3 | 86 | 23 | 57 | 2.5 | 46 |
| 9 | F | 55 | Renal cancer | 265 | 39 | 176 | 4.5 | 110 | 21 | 47 | 2.2 | 59 |
| 10 | M | 60 | Renal cancer | 154 | 42 | 103 | 2.4 | 107 | 22 | 56 | 2.4 | 31 |
| 11 | M | 55 | Colon cancer | 238 | 41 | 175 | 4.2 | 127 | 19 | 71 | 3.7 | 47 |
| 12 | M | 72 | Colon cancer | 215 | 35 | 159 | 4.5 | 140 | 26 | 72 | 3.4 | 35 |
| 13 | M | 57 | Colon cancer | 202 | 34 | 107 | 3.1 | 109 | 19 | 41 | 2.2 | 46 |
| 14 | M | 49 | Colon cancer | 209 | 39 | 145 | 3.7 | 85 | 16 | 58 | 3.6 | 59 |

other factors, including leukocyte proliferation (Rosenberg et al., 1987) and cortisol rise (Denicoff et al., 1989) induced by IL-2, would not be enough to explain the rapid and very pronounced fall in cholesterol levels, which occurs within few days from the start of IL-2 therapy, and precedes leukocyte proliferation.

The progressive decline with time in IL-2 cholesterol-lowering activity, observed in this study despite the continuous IL-2 injection and not shown by Wilson et al. (1989) with a shorter period of administration, might depend on a possible down-regulation of IL-2 macrophage receptors, determined by the prolonged administration of IL-2. The difference between our results and those referred by Wilson et al. (1989), who described a more rapid return to baseline values with IL-2 interruption, could be due to differences in doses, route and schedule of IL-2 therapy.

The evidence that IL-2 affects cholesterol metabolism would suggest a possible role of IL-2, which endogenous availability decreases with age (Saadeh et al., 1986), in the pathogenesis of atherosclerosis, as recently proposed for other cytokines (Ross, 1986). If future studies will confirm also in non-oncologic hypercholesterolemic patients its cholesterol-lowering activity, clinical trials will be justified to investigate the possible use of IL-2 in the treatment of cholesterol metabolism disorders.

### References

ATZPODIEN, J., KORFER, A., FRANKS, C.R., POLIWODA, H. & KIRCHNER, H. (1990). Home therapy with recombinant interleukin-2 and interferon-α 2b in advanced human malignancies. *Lancet*, **335**, 1509.

CAI, H.J., HE, Z.G. & DING, Y.N. (1988). Effects of monocyte macrophage stimulation on hepatic lipoprotein receptors. *Biochem. Biophys. Acta*, **959**, 334.

CHAMBRIER, C., MERCATELLO, A., TOGNET, E. & 6 others (1990). Hormonal and metabolic effects of chronic interleukin-2 infusion in cancer patients. *J. Biol. Response Mod.*, **9**, 215.

DENICOFF, K.D., DURKIN, T.M., LOTZE, M.T. & 5 others (1989). The neuroendocrine effects of interleukin-2 treatment. *J. Clin. Endocrinol. Metab.*, **69**, 402.

FOGELMAN, A.M., SEAGER, J., GROOPMAN, J.E. & 4 others (1983). Lymphokines secreted by an established lymphocyte line modulate receptor-mediated endocytosis in macrophages derived from human monocytes. *J. Immunol.*, **131**, 2368.

FOGELMAN, A.M., SEAGER, J., HABERLAND, M.E. & 4 others (1982). Lymphocyte-conditioned medium protects human monocyte-macrophages from cholesterol ester accumulation. *Proc. Natl Acad. Sci. USA*, **79**, 922.

GOLDSTEIN, J.L., HO, Y.K., BASY, S.K. & 4 others (1979). Binding site of macrophages that mediates uptake and degradation of acetylated low density lipoprotein, producing massive cholesterol deposition. *Proc. Natl Acad. Sci. USA*, **76**, 333.

GORDON, T., CASTELLI, W.P., HJORTLAND, M.C., KANNEL, W.B. & DAWBER, T.R. (1979). High density lipoprotein as a protective factor against coronary heart disease. *Am. J. Med.*, **62**, 707.

LEE, R.E., LOTZE, M.T., SKIBBER, J.M. & 7 others (1989). Cardiorespiratory effects of immunotherapy with interleukin-2. *J. Clin. Oncol.*, **7**, 7.

MALKOVSKY, M. & SONDEL, P.M. (1987). Interleukin-2 and its receptor: structure, function and therapeutic potential. *Blood Rev.*, **1**, 254.

MICHALEVICZ, R., CAMPANA, D., KATZ, F., JANOSSY, G. & HOFFBRAND, A.V. (1988). Recombinant interleukin 2 and anti-Tac influence the growth of enriched multipotent hemopoietic progenitors: proposed hypotheses for different responses in early and late progenitors. *Leukaemia Res.*, **12**, 113.

NEDWIN, G.E., SVEDFESKY, L.P., BRINGMAN, T.S. & 5 others (1985). Effect of interleukin 2, interferon-gamma, and mitogens on the production of tumor necrosis factor alpha and beta. *J. Immunol.*, **135**, 2492.

NIMER, S.D., CHAMPLIN, R.E. & GOLDE, D.W. (1988). Serum cholesterol-lowering activity of granulocyte-macrophage colony-stimulating factor. *JAMA*, **260**, 3297.

ROSENBERG, S.A., LOTZE, M.T., MUUL, L.M. & 10 others (1987). A progressive report on the treatment of 157 patients with advanced cancer using lymphokine-activated killer cells and interleukin-2 or high-dose interleukin-2 alone. *N. Engl. J. Med.*, **316**, 889.

ROSS, R. (1986). The pathogenesis of atherosclerosis: an update. *N. Engl. J. Med.*, **314**, 488.

SAADEH, C., AUZENNE, C., NELSON, D. & ORSON, F. (1986). Sera from the aged contain higher levels of IL-2 receptor compared to young adults. *Fed. Proc.*, **45**, 378.

SHERMAN, M.L., SPRIGGS, D.R., ARTHUR, K.A., IMAMURA, K., FREI, E. & KUFE, D.W. (1988). Recombinant human tumor necrosis factor administered as a five-day continuous infusion in cancer patients: phase I toxicity and effects on lipid metabolism. *J. Clin. Oncol.*, **6**, 344.

TOBERT, J.A. (1987). New developments in lipid-lowering therapy: the role of inhibitors of hydroxymethylglutaryl-coenzyme A reductase. *Circulation*, **76**, 534.

WILSON, D.E., BIRCHFIELD, G.R., HEJAZI, J.S., WARD, J.H. & SAMLOWSKI, W.E. (1989). Hypocholesterolemia in patients treated with recombinant interleukin-2: appearance of remnant-like lipoproteins. *J. Clin. Oncol.*, **7**, 1573.


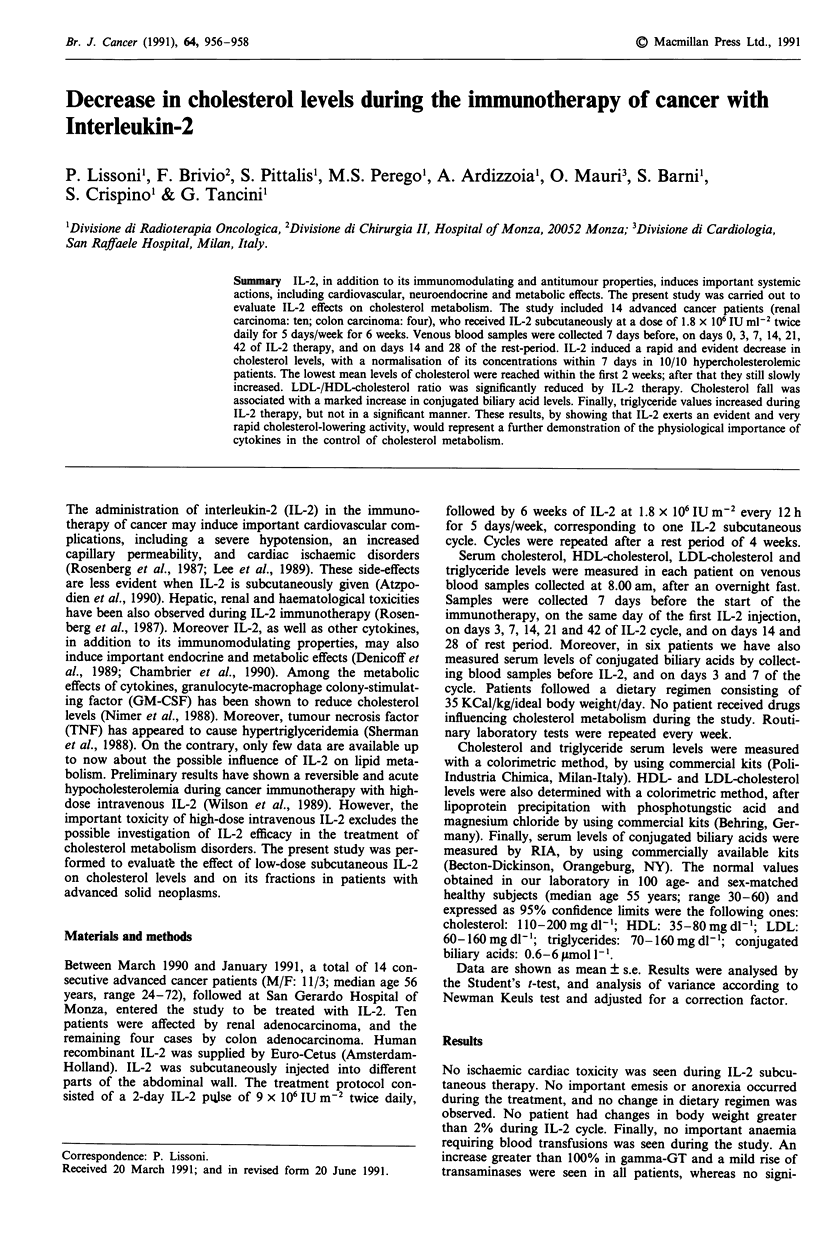

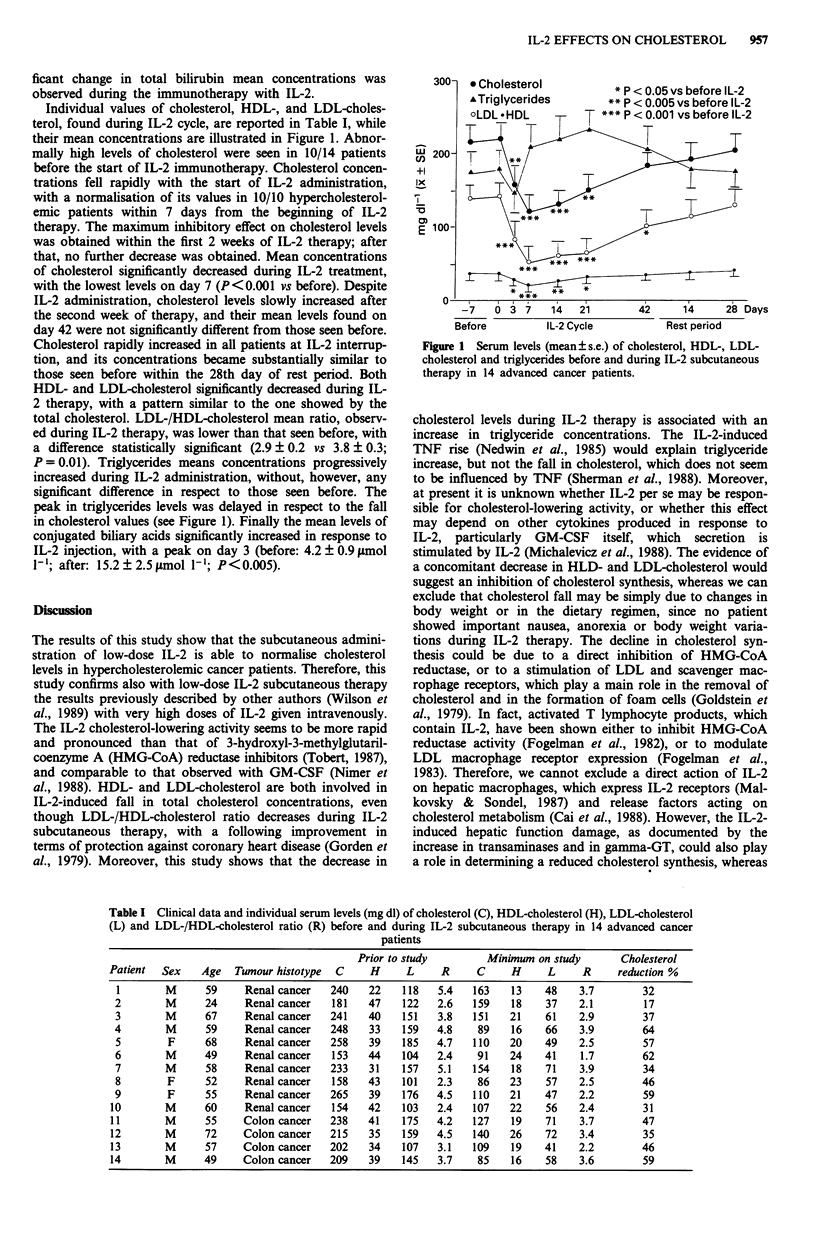

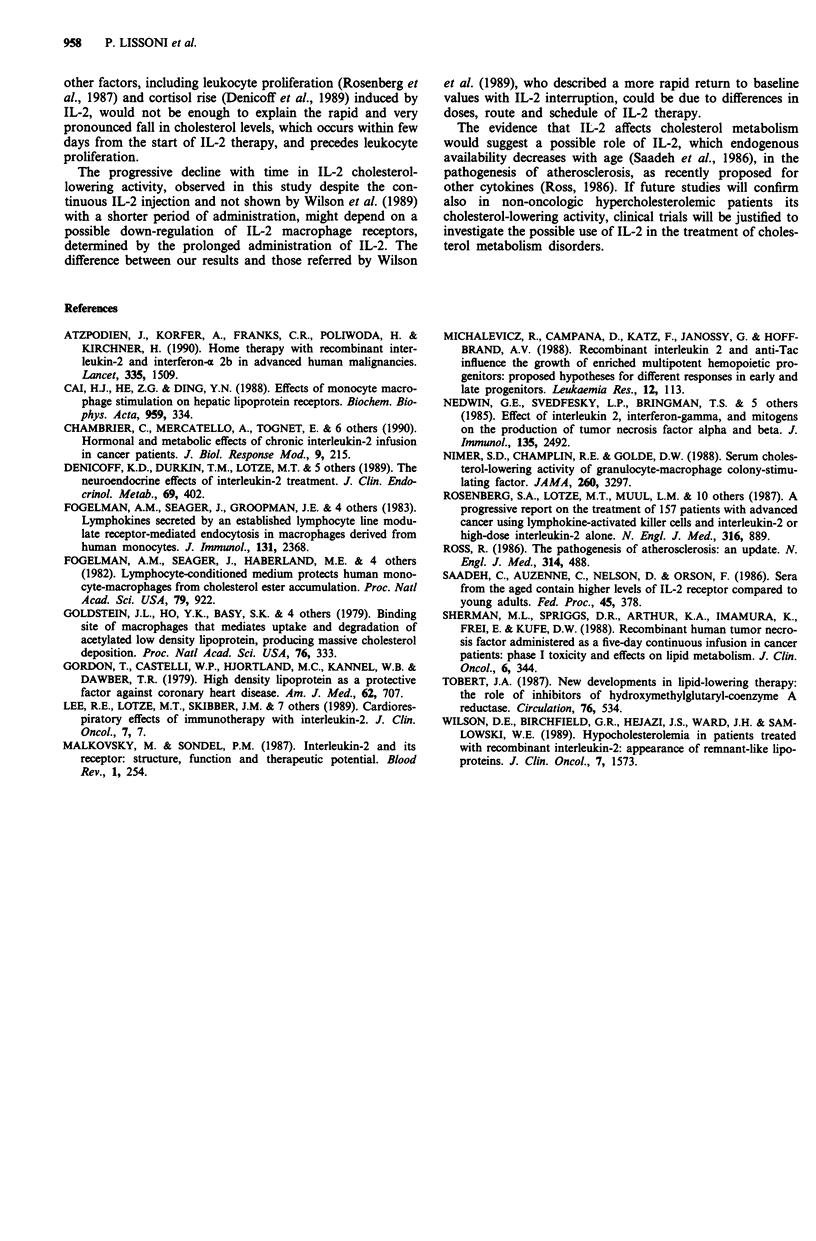


## References

[OCR_00298] Atzpodien J., Körfer A., Franks C. R., Poliwoda H., Kirchner H. (1990). Home therapy with recombinant interleukin-2 and interferon-alpha 2b in advanced human malignancies.. Lancet.

[OCR_00304] Cai H. J., He Z. G., Ding Y. N. (1988). Effects of monocyte macrophages stimulation on hepatic lipoprotein receptors.. Biochim Biophys Acta.

[OCR_00316] Denicoff K. D., Durkin T. M., Lotze M. T., Quinlan P. E., Davis C. L., Listwak S. J., Rosenberg S. A., Rubinow D. R. (1989). The neuroendocrine effects of interleukin-2 treatment.. J Clin Endocrinol Metab.

[OCR_00321] Fogelman A. M., Seager J., Groopman J. E., Berliner J. A., Haberland M. E., Edwards P. A., Golde D. W. (1983). Lymphokines secreted by an established lymphocyte line modulate receptor-mediated endocytosis in macrophages derived from human monocytes.. J Immunol.

[OCR_00325] Fogelman A. M., Seager J., Haberland M. E., Hokom M., Tanaka R., Edwards P. A. (1982). Lymphocyte-conditioned medium protects human monocyte-macrophages from cholesteryl ester accumulation.. Proc Natl Acad Sci U S A.

[OCR_00331] Goldstein J. L., Ho Y. K., Basu S. K., Brown M. S. (1979). Binding site on macrophages that mediates uptake and degradation of acetylated low density lipoprotein, producing massive cholesterol deposition.. Proc Natl Acad Sci U S A.

[OCR_00337] Gordon T., Castelli W. P., Hjortland M. C., Kannel W. B., Dawber T. R. (1977). High density lipoprotein as a protective factor against coronary heart disease. The Framingham Study.. Am J Med.

[OCR_00347] Malkovský M., Sondel P. M. (1987). Interleukin 2 and its receptor: structure, function and therapeutic potential.. Blood Rev.

[OCR_00359] Nedwin G. E., Svedersky L. P., Bringman T. S., Palladino M. A., Goeddel D. V. (1985). Effect of interleukin 2, interferon-gamma, and mitogens on the production of tumor necrosis factors alpha and beta.. J Immunol.

[OCR_00365] Nimer S. D., Champlin R. E., Golde D. W. (1988). Serum cholesterol-lowering activity of granulocyte-macrophage colony-stimulating factor.. JAMA.

[OCR_00370] Rosenberg S. A., Lotze M. T., Muul L. M., Chang A. E., Avis F. P., Leitman S., Linehan W. M., Robertson C. N., Lee R. E., Rubin J. T. (1987). A progress report on the treatment of 157 patients with advanced cancer using lymphokine-activated killer cells and interleukin-2 or high-dose interleukin-2 alone.. N Engl J Med.

[OCR_00376] Ross R. (1986). The pathogenesis of atherosclerosis--an update.. N Engl J Med.

[OCR_00385] Sherman M. L., Spriggs D. R., Arthur K. A., Imamura K., Frei E., Kufe D. W. (1988). Recombinant human tumor necrosis factor administered as a five-day continuous infusion in cancer patients: phase I toxicity and effects on lipid metabolism.. J Clin Oncol.

[OCR_00392] Tobert J. A. (1987). New developments in lipid-lowering therapy: the role of inhibitors of hydroxymethylglutaryl-coenzyme A reductase.. Circulation.

[OCR_00399] Wilson D. E., Birchfield G. R., Hejazi J. S., Ward J. H., Samlowski W. E. (1989). Hypocholesterolemia in patients treated with recombinant interleukin-2: appearance of remnant-like lipoproteins.. J Clin Oncol.

